# Unilateral lifebuoy cataract: A case report

**DOI:** 10.1097/MD.0000000000039359

**Published:** 2024-08-16

**Authors:** Yuka Koshiishi, Mayumi Nagata, Hiroyuki Matsushima, Sakae Ito, Shigenari Suzuki, Haruka Matsumoto, Akihiko Okayasu, Tadashi Senoo

**Affiliations:** a Department of Ophthalmology, Dokkyo Medical University Hospital, Tochigi, Japan.

**Keywords:** congenital cataract, lifebuoy cataract, Nd, unilateral cataract, YAG laser

## Abstract

**Rationale:**

Lifebuoy cataract is a rare congenital condition characterized by lens thinning. Due to its rarity, detailed treatment reports and standardized surgical approaches are limited. This study aims to enhance the current body of knowledge by presenting comprehensive case reports and describing surgical techniques for the treatment of lifebuoy cataracts.

**Patient concerns:**

A 14-year-old boy was diagnosed with a congenital cataract in his right eye at the age of 9, which was left untreated. The patient visited our hospital due to progressive visual impairment.

**Diagnoses:**

The visual acuity of the right eye was counting fingers at 30 cm. The uncorrected visual acuity of the left eye was 20/100, whereas the best corrected visual acuity was 20/20. The intraocular pressures were 18 mm Hg (left eye) and 20 mm Hg (right eye). Slit-lamp microscopy revealed central calcification of the lens capsule in the right eye and slightly opaque cortical tissue in the periphery, with no observable lens nucleus. Anterior segment optical coherence tomography (CASIA2, TOMEY, Nagoya, Japan) of the right eye showed fused anterior and posterior capsules and an absence of the lens nucleus, leading to a diagnosis of lifebuoy cataract.

**Interventions:**

Cataract surgery was performed on the right eye. Following a 2.4-mm sclerocorneal incision and trypan blue staining, continuous curvilinear capsulorrhexis was performed around the central opacity. The surrounding cortex was removed using irrigation and aspiration, while a viscoelastic agent was injected between the central calcified membrane and the posterior capsule. The membranous tissue was carefully peeled away and removed using forceps. Despite residual posterior capsular opacification, posterior capsulotomy was not performed due to concerns about vitreous prolapse. The intraocular lens was fixed within the capsule. Ten days post-surgery, the remaining posterior capsular opacification was treated with neodymium-doped yttrium aluminum garnet laser capsulotomy.

**Outcomes:**

The uncorrected visual acuity and best corrected visual acuity of the right eye improved to 20/100 and 20/50, respectively.

**Lessons:**

This case report demonstrates a successful surgical approach for a lifebuoy cataract, highlighting its unique morphology and the need for careful, specialized techniques. These findings aim to guide ophthalmologists in managing this rare condition, potentially improving patient care.

## 1. Introduction

Congenital cataracts develop at birth or during early childhood and interfere with normal visual development, as well as overall development.^[1,2]^ Therefore, early diagnosis and prompt therapeutic intervention is necessary.^[3,4]^

Various forms of congenital cataracts have been reported, including interlaminar cataracts, nuclear cataracts, suture cataracts, total cataracts, and posterior conical lenses.^[5]^ However, lifebuoy cataracts have been reported as rare.^[6]^ Lifebuoy cataracts are characterized by the absence of a lens nucleus and the formation of a ring-shaped cortical opacification,^[6,7]^ requiring a different surgical technique from that of regular cataract surgery. However, few studies have reported clinical characteristics, surgical techniques, and postoperative course. Herein, we report a case of unilateral lifebuoy cataracts with favorable treatment outcomes.

## 2. Case presentation

The patient was a 14-year-old boy with the chief complaint of decreased visual acuity in the right eye. At the age of 9 years, he was diagnosed with a congenital cataract in the right eye. However, his father had been transferred overseas, leaving the cataract untreated. The patient visited our hospital because of progressive visual impairment of the right eye over the past several months. His medical and family histories were unremarkable.

**Figure 1.**
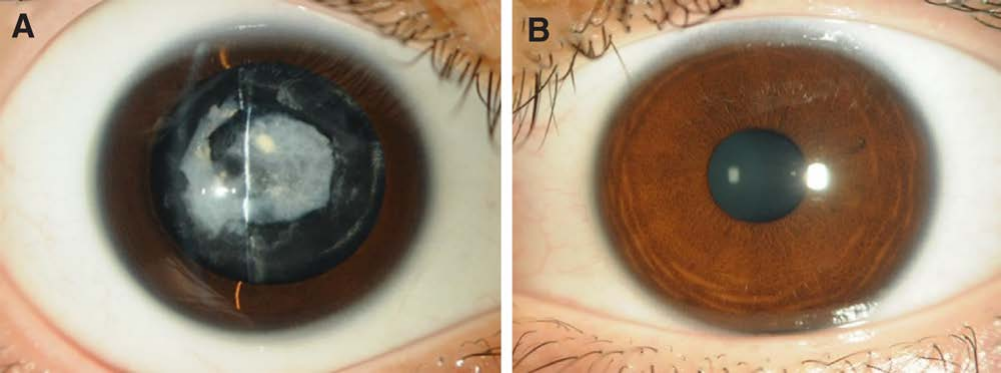
Preoperative findings of the anterior segment. (A) Right eye. (B) Left eye.

**Figure 2.**
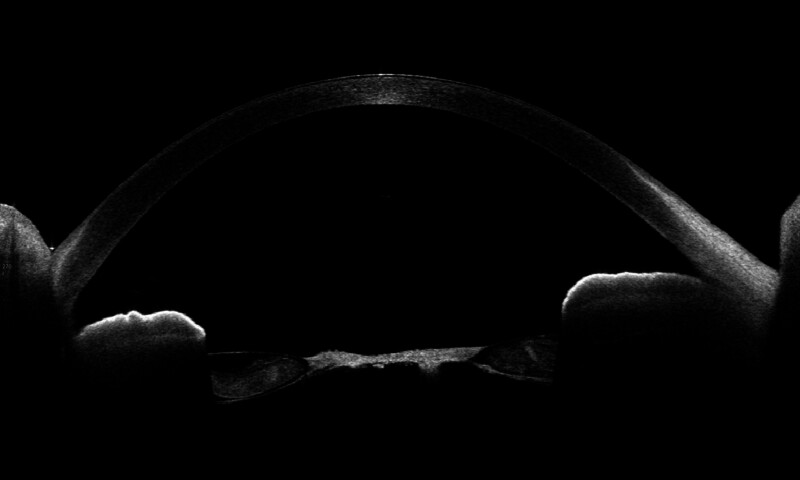
Preoperative AS-OCT findings. AS-OCT = anterior segment optical coherence tomography.

At the first visit, the best corrected visual acuity (BCVA) in the right eye was counting fingers (CF) at 30 cm. The uncorrected visual acuity (UCVA) of the left eye was 20/100, BCVA 20/20 (with −2.5 sph −0.75 cyl 180°). Corneal astigmatism was measured using a corneal shape analyzer (CASIA2, TOMEY, Nagoya, Japan) and was −4.28 D in the right eye and −1.54 D in the left eye. The intraocular pressure was 18 mm Hg in the right eye and 20 mm Hg in the left eye. The number of corneal endothelial cells was 3293 cells/mm^2^ in the right eye and 3075 cells/mm^2^ in the left eye. The axial length (UD-8000, TOMEY, Nagoya, Japan) was 24.26 mm for the right eye and 25.05 mm for the left eye, and the eye position was normal. Slit-lamp microscopy revealed a calcified lens capsule in the center and a slightly opacified cortical tissue in the periphery; however, no lens nuclei were observed (Fig. 1A). The fundus of the right eye was difficult to observe because of the cataract; however, the electroretinogram was normal. No abnormal findings were observed in the left anterior segment (Fig. 1B) or fundus. Anterior segment optical coherence tomography (AS-OCT, CASIA2, TOMEY, Nagoya, Japan) showed that the anterior and posterior capsules of the lens were fused, and the lens nucleus was absent (Fig. 2). Therefore, a lifebuoy cataract was diagnosed.

**Figure 3.**
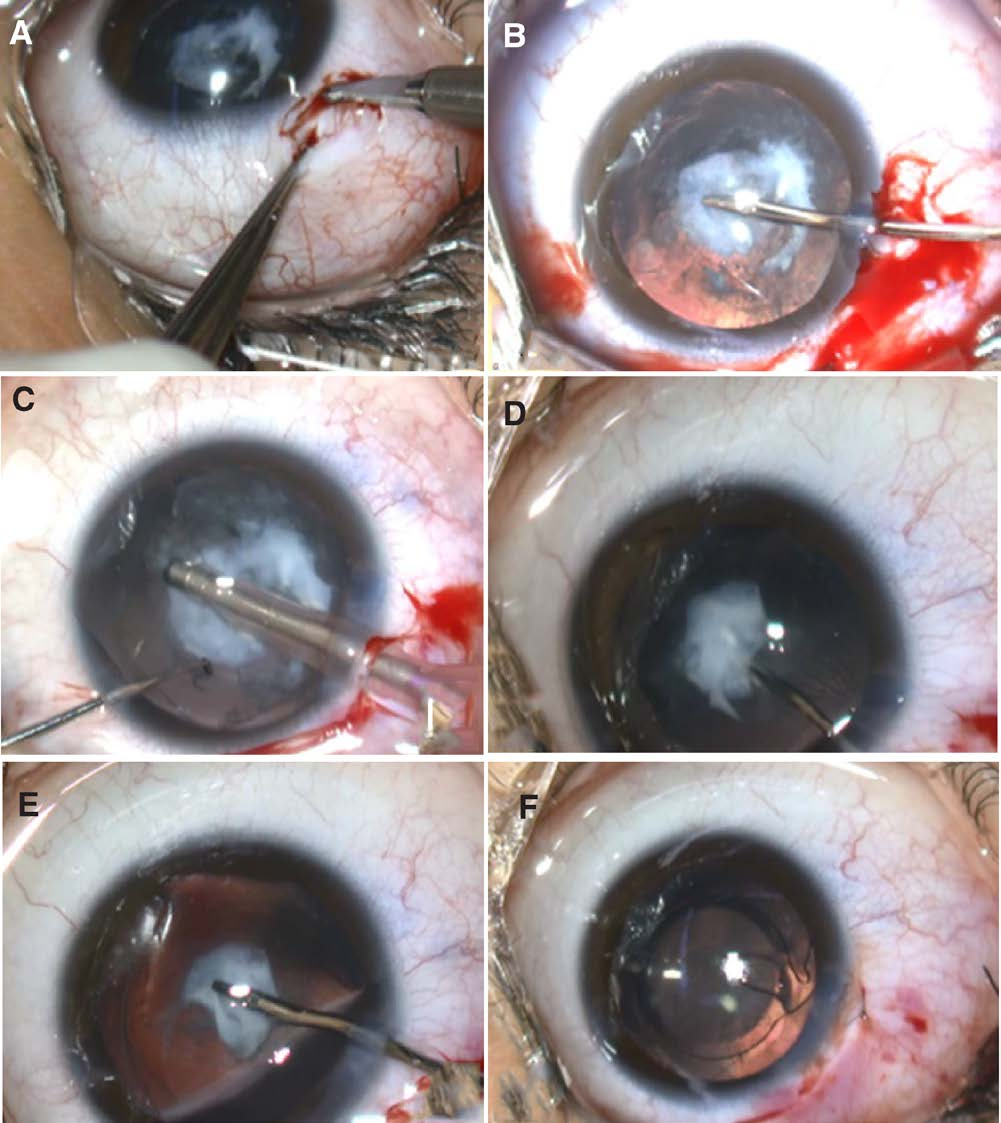
Intraoperative findings.

After sub-tenon anesthesia with xylocaine and 2% adrenaline (Sandoz K.K., Tokyo, Japan), a 2.4-mm sclerocorneal incision was made at the 11 o’clock position (Fig. 3A). After creating side ports at the 10 o’clock and 2 o’clock sites, 0.1% diluted trypan blue^[8]^ (Sigma-Aldrich Corporation, MO) was injected into the anterior chamber to stain the anterior capsule, and BSS was perfused into the anterior chamber through the side ports for irrigation. The soft-shell technique was performed using Healon 1% ophthalmic viscoelastic substance (AMO, Hyogo, Japan) and Shellgan 0.5 ophthalmic viscoelastic substance (Seikagaku Corporation, Tokyo, Japan) as ophthalmic viscosurgical devices (OVD). Subsequently, continuous curvilinear capsulorrhexis (CCC) was performed using anterior capsular forceps. Given the strong opacity with calcification in the center of the anterior capsule, CCC was completed by passing the incision edge around the opacity (Fig. 3B). Phacoemulsification was performed using Constellation (Alcon, Tokyo, Japan), an ophthalmic microsurgical system. First, to prevent damage to the posterior capsule, hydrodissection was not performed, and the surrounding cortex within the lens capsule was removed by suction using an irrigation and aspiration (I/A) handpiece (Fig. 3C). Next, we attempted to remove the calcified membranous tissue in the center using anterior capsule forceps; however, this was difficult because the tissue adhered to the posterior capsule. While injecting 1% ophthalmic viscoelastic agent (AMO, Hyogo, Japan) into the gap between the membranous tissue and the posterior capsule, it was carefully dissected from the posterior capsule (Fig. 3D) and removed with anterior capsule forceps (Fig. 3E). Posterior capsular opacification (PCO) due to calcification was observed, but posterior capsulotomy was not performed because of concerns about vitreous prolapse during surgery. An intraocular lens (IOL, Tecnis^®^OptiBlue^®^, +18.0D, AMO, Hyogo, Japan) was inserted into the lens capsule (Fig. 3F), and the surgery was completed.

**Figure 4.**
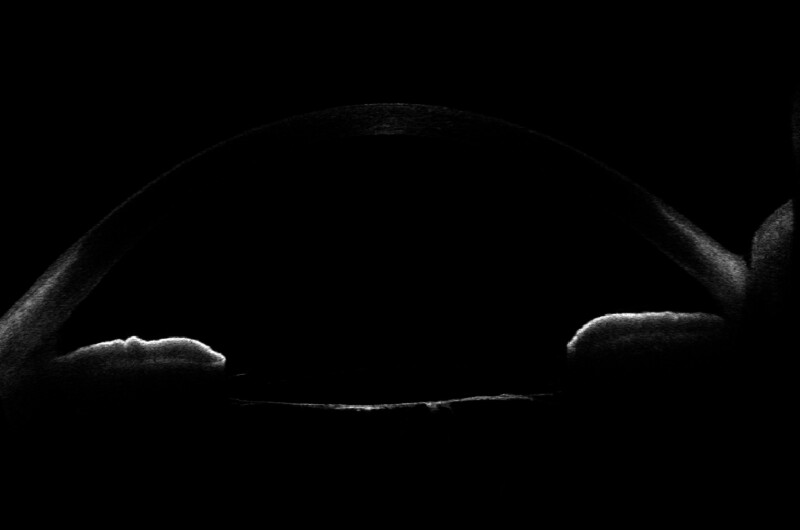
Postoperative AS-OCT findings. AS-OCT = anterior segment optical coherence tomography.

**Figure 5.**
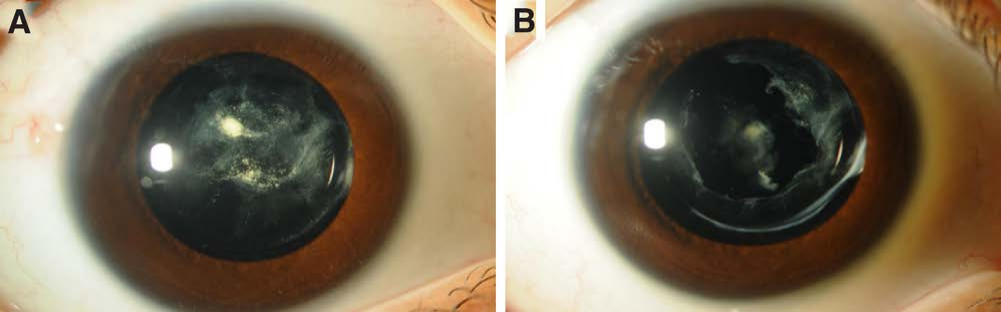
Photograph of the postoperative anterior segment. (A) Findings after right eye cataract surgery. (B) Photograph after right eye YAG laser posterior capsulotomy.

The BCVA of the right eye on the day after surgery was 20/100 (uncorrectable), and intracapsular fixation of the IOL was confirmed by AS-OCT (Fig. 4). A posterior capsulotomy was performed using a YAG laser (Fig. 5B) to remove the remaining posterior capsule opacity ten days after the surgery (Fig. 5A). The neodymium-doped yttrium aluminum garnet (Nd:YAG) laser was set at 2.0 mJ, and 232 shots were used to irradiate the posterior capsule. After irradiation, nonsteroidal anti-inflammatory eye drops (0.1 bromfenac sodium ophthalmic solution; Nissin, Yamagata, Japan) were used to manage inflammation. UCVA improved to 20/100, BCVA to 20/50 (with + 3.25sph −4.9cyl 180°) 1 month postoperatively, and no significant changes were observed. The intraocular pressure in the right eye was 16 mm Hg, and the number of corneal endothelial cells in the right eye was 3276 cells/mm^2^. Fundus examination revealed no abnormalities in the right eye. The patient currently attends follow-up appointments every 3 months.

All figures were created for this article.

## 3. Discussion

We encountered a case of lifebuoy cataract, which is a rare congenital cataract. Lifebuoy cataracts are also called disk-shaped, ring, annular, dumbbell-shaped, or umbilical cataracts. Regarding its pathogenesis, lifebuoy cataract is considered an embryological disorder that occurs during the fifth week of life, when the fetal nucleus does not develop but shrinks, becomes calcified, and remains attached to the lens capsule.^[7]^ In general, it is reported to be binocular and is often accompanied by ocular complications such as lens deviation, anterior segment abnormalities such as lens coloboma, and foveal hypoplasia.^[9]^ In Japan, Tsuji et al^[10]^ reported thinning cataracts associated with congenital microcoria in 2014. However, the patient had no history of ocular or systemic diseases other than a congenital cataract in his right eye that was noted at another hospital when he was 9 years old. Thereafter, no other abnormal findings were found on examination. Therefore, this is a rare case of a unilateral lifebuoy cataract. Additionally, it is unclear when lifebuoy cataracts were first recognized because medical information on infancy is difficult to obtain, but it has been reported that congenital cataracts that develop in infancy gradually progress.^[11]^ In this case, the patient was aware that the visual acuity in his right eye had worsened, and it is thought that the opacity of the lens increased gradually.

Maamouri et al^[6]^ reported the clinical findings and surgical technique involving a 42-year-old patient with a lifebuoy cataract. In that case, the lens nucleus was not found, and the anterior and posterior capsules of the lens were fused. It was difficult to remove the membranous calcified tissue; it was finally removed through a posterior capsulotomy, and the IOL was fixed in the ciliary sulcus. Maamouri et al suggested that patients with lifebuoy cataracts should be prepared using vitrectomy equipment and triamcinolone before surgery. Posterior capsulotomy is often performed with a vitreous cutter during cataract surgery to manage PCOs,^[12,13]^ and its safety and favorable long-term prognosis have been reported.^[14]^ However, the degree of adhesion varies depending on the case. In our case, there was a gap between the membranous calcified tissue and the posterior capsule, and we managed to inject the OVD into this space and dissect the membranous tissue. We also chose not to perform posterior capsulotomy intraoperatively as a postoperative Nd:YAG laser capsulotomy could be performed postoperatively, as the patient was already 14 years old. The advantages of avoiding surgical invasiveness due to anterior vitrectomy after posterior capsulotomy and ensuring secure fixation of the IOL within the capsule were also considered. The irradiation power of the Nd:YAG laser was set to 2.0 mJ. The opacity of the posterior capsule was strong, requiring a large number of irradiations.

The critical period for visual development in children is 6 weeks of age. It is said that the development of stimulus-blocking amblyopia, strabismus, and nystagmus can be prevented by removing unilateral congenital cataracts by 4 to 6 weeks after birth and bilateral congenital cataracts by 6 to 8 weeks after birth.^[4]^ Although there is no need for immediate surgery for cases of partial cataracts, those with strong astigmatism or decreased contrast sensitivity have a poor visual prognosis.^[15]^ This patient did not have severe amblyopia, probably due to the partial cataract, but the final postoperative BCVA was 20/50, suggesting a limit to the improvement of visual function.

This study has some limitations. First, it reported only one case of unilocular lifebuoy cataracts, which may not be representative of this disease as it is generally considered bilateral. Furthermore, although the patient reported no family history or systemic disease complications during the interview, no genetic testing or detailed systemic evaluation was performed. Thus, this study is insufficient to establish the general characteristics and treatment of lifebuoy cataracts. Second, because of the relatively short postoperative follow-up period, we lack sufficient information on long-term visual improvement, potential complications, and long-term effects on visual development and learning ability. It has been reported that posterior capsule opacification recurs due to the proliferation of lens epithelial cells after YAG laser treatment in children with congenital cataracts.^[16]^ Therefore, careful follow-up is required in the future.

In this case, we successfully treated a 14-year-old boy with unilateral lifebuoy cataracts, achieving favorable outcomes. Visual acuity improved from CF at 30 cm preoperatively to a BCVA of 20/50 1 month postoperatively. Despite the complex surgical procedure, we completed the operation without major complications and successfully fixed the IOL within the capsule. Ten days post-surgery, we performed Nd:YAG laser capsulotomy to address residual PCO. The patient continues to attend follow-up appointments every 3 months, with stable vision to date. However, due to the nature of congenital cataracts, complete visual function recovery was limited, with final BCVA remaining at 20/50. Long-term follow-up is necessary, particularly to monitor for PCO recurrence and binocular visual development. This case provides a valuable report on the surgical approach and outcomes for rare lifebuoy cataracts, contributing to the limited body of knowledge on this condition.

Furthermore, prompt diagnosis, follow-up by ophthalmologists and pediatricians, and active intervention by school and community pediatric vision support services^[17]^ are important to promote good visual development and prevent amblyopia in patients with congenital cataracts, such as in the present case.

## Acknowledgments

We would like to thank Editage (www.editage.com) for English language editing.

## Author contributions

**Conceptualization:** Yuka Koshiishi.

**Software:** Yuka Koshiishi.

**Validation:** Yuka Koshiishi, Mayumi Nagata.

**Writing – original draft:** Yuka Koshiishi.

**Writing – review & editing:** Yuka Koshiishi, Mayumi Nagata, Shigenari Suzuki, Haruka Matsumoto.

**Formal analysis:** Mayumi Nagata.

**Methodology:** Hiroyuki Matsushima.

**Resources:** Hiroyuki Matsushima.

**Visualization:** Hiroyuki Matsushima, Akihiko Okayasu.

**Data curation:** Sakae Ito.

**Supervision:** Tadashi Senoo.
